# Proteomic Diversity of the Sea Anemone *Actinia fragacea*: Comparative Analysis of Nematocyst Venom, Mucus, and Tissue-Specific Profiles

**DOI:** 10.3390/md23020079

**Published:** 2025-02-11

**Authors:** Ricardo Alexandre Barroso, Tomás Rodrigues, Alexandre Campos, Daniela Almeida, Francisco A. Guardiola, Maria V. Turkina, Agostinho Antunes

**Affiliations:** 1 CIIMAR/CIMAR—Interdisciplinary Centre of Marine and Environmental Research, University of Porto, Terminal de Cruzeiros do Porto de Leixões, Av. General Norton de Matos, s/n, 4450-208 Porto, Portugal; barrosoalex98@gmail.com (R.A.B.); tomasfcr.porto@gmail.com (T.R.); amoclclix@gmail.com (A.C.); 2 Department of Biology, Faculty of Sciences, University of Porto, Rua do Campo Alegre 687, 4169-007 Porto, Portugal; 3 Department of Zoology and Physical Anthropology, Faculty of Biology, University of Murcia, Campus of International Excellence, Campus Mare Nostrum, 30100 Murcia, Spain; daniela.martins@um.es; 4 Immunobiology for Aquaculture Group, Department of Cell Biology and Histology, Faculty of Biology, Regional Campus of International Excellence “Campus Mare Nostrum”, University of Murcia, 30100 Murcia, Spain; faguardiola@um.es; 5 Department of Biomedical and Clinical Sciences, Faculty of Medicine and Clinical Sciences, Linköping University, 581 83 Linköping, Sweden; maria.turkina@liu.se

**Keywords:** sea anemones, Cnidaria, proteomics, toxins, antimicrobial peptides (AMPs)

## Abstract

Sea anemones (Actiniaria, Cnidaria) are promising targets for biomedical research, as they produce unique bioactive compounds, including toxins and antimicrobial peptides (AMPs). However, the diversity and mechanisms underlying their chemical defenses remain poorly understood. In this study, we investigate the proteomic profiles of the unexplored sea anemone *Actinia fragacea* by analyzing its venom nematocyst extract, tissues, and mucus secretion. A total of 4011 different proteins were identified, clustered into 3383 protein groups. Among the 83 putative toxins detected, actinoporins, neurotoxins, and phospholipase A2 were uncovered, as well as two novel zinc metalloproteinases with two specific domains (ShK) associated with potassium channel inhibition. Common Gene Ontology (GO) terms were related to immune responses, cell adhesion, protease inhibition, and tissue regeneration. Furthermore, 1406 of the 13,276 distinct peptides identified were predicted as potential AMPs, including a putative Aurelin-like AMP localized within the nematocysts. This discovery highlights and strengthens the evidence for a cnidarian-exclusive Aurelin peptide family. Several other bioactive compounds with distinctive defense functions were also detected, including enzymes, pattern recognition proteins (PRPs), and neuropeptides. This study provides the first proteome map of *A. fragacea*, offering a critical foundation for exploring novel bioactive compounds and valuable insights into its molecular complexity.

## 1. Introduction

The phylum Cnidaria, recognized as the sister group of Bilateria [1], represents the oldest extant lineage of venomous animals [2,3,4,5], encompassing approximately 11,000 described species [6]. This group exhibits remarkable diversity in morphology, lifecycles, ecology, and development processes, being established by three major clades—the sessile Anthozoa (e.g., sea anemones, stony corals, and sea fans), the free-living Medusozoa (e.g., jellyfish and Hydrozoa), and the microscopic endoparasites Myxozoa [7,8,9]. These animals possess specialized stinging cells distributed along their body, named cnidocytes, with harpoon-like large secretory organelles—nematocysts—that have an established role in venom delivery, being discharged upon contact for prey capture and defense against predators [2,10,11]. Cnidaria venom is constituted by a combination of several peptides and other bioactive molecules, collectively referred to as toxins. Known peptide toxins from Cnidaria include enzymes (e.g., phospholipase A2, metalloproteinases), membrane-active toxins (e.g., jellyfish toxins, actinoporins, and hydralysins), neurotoxins (e.g., sodium- and potassium-channel inhibitory toxins and SCRiPs—small cysteine-rich proteins) and protease inhibitors [12,13,14,15,16]. Moreover, cnidarians rely heavily on their innate immune systems through the production of AMPs, as they thrived in challenging environments for hundreds of millions of years with high microbial and viral loads (approximately 10^6^ bacteria/mL and 10^9^ viruses/mL of seawater [17]) and without adaptative immune responses [18,19,20]. Some uncovered AMPs include Aurelin [21] from the scyphoid jellyfish *Aurelia aurita* and Damicornin [21] from the stony coral *Pocillopora damicornis*, with potent antibacterial and antifungal properties.

Sea anemones (order Actiniaria) are generally large, solitary soft-bodied polyps usually attached to rocks or other suitable substrata, with approximately 1350 species estimated to exist worldwide [22]. They are distributed from tropical to polar regions, occupying several habitats from the intertidal oceanic zones to depths of more than 10,000 m [11]. Sea anemones possess vital roles in marine ecosystems. They act as predators of several organisms, including crustaceans, fish, and mollusks, which are caught with tentacles, paralyzed and secured by the cnidae, and carried to the mouth. Mutualistic interactions are also common with hermit crabs and fish of the genus *Amphiprion* (clownfish) [22]. Sea anemones account for most of the toxins uncovered in Cnidaria, which have been identified to date by traditional protein analyses [12]. Also, their ecological function can be inferred by their location in the sea anemone tissues. Neurotoxins and membrane-active toxins are more common in the tentacles and epidermis, supporting prey immobilization and predator deterrence. Enzymatic toxins support digestive roles in the gastrodermis and mesenteric filaments [3,23,24,25]. Sea anemones also have additional structures from other anthozoans. Acrorhagi are found within the Actiniidae family, inflatable organs located below the tentacles with roles in intraspecific aggression. Acrorhagins from *Actinia equina* were the first described toxins from the acrorhagi, showing lethality in crabs [26]. Additionally, acontia is a structure unique to specific lineages of the superfamily Metridioidea, constituted from long thread-like structures formed from the mesenterial filaments with a possible role in defense [27]. Depending on the species, their toxins may be localized in both nematocysts and ectodermal gland cells [28]. Interestingly, certain neurotoxins produced by sea anemones exhibit antimicrobial properties, highlighting a dual role in their survival. These toxins facilitate prey capture and defend against bacterial infections that could result from injuries to their tentacles [29,30].

The strawberry anemone *A. fragacea* [31], with yellow or green spots displayed in its column, is genetically distinct from the most abundant *A. equina* in the northern coasts of Portugal, which shows a high degree of polymorphism. Compared with *A. equina*, *A. fragacea* lacks viviparity, is larger (up to 10 cm in diameter), and is mainly found solitary [32,33]. Five pore-forming toxins (actinoporins) causing hemolysis are known from *A. fragacea*, corresponding to the Fragaceatoxins [33,34].

**Figure 1:**
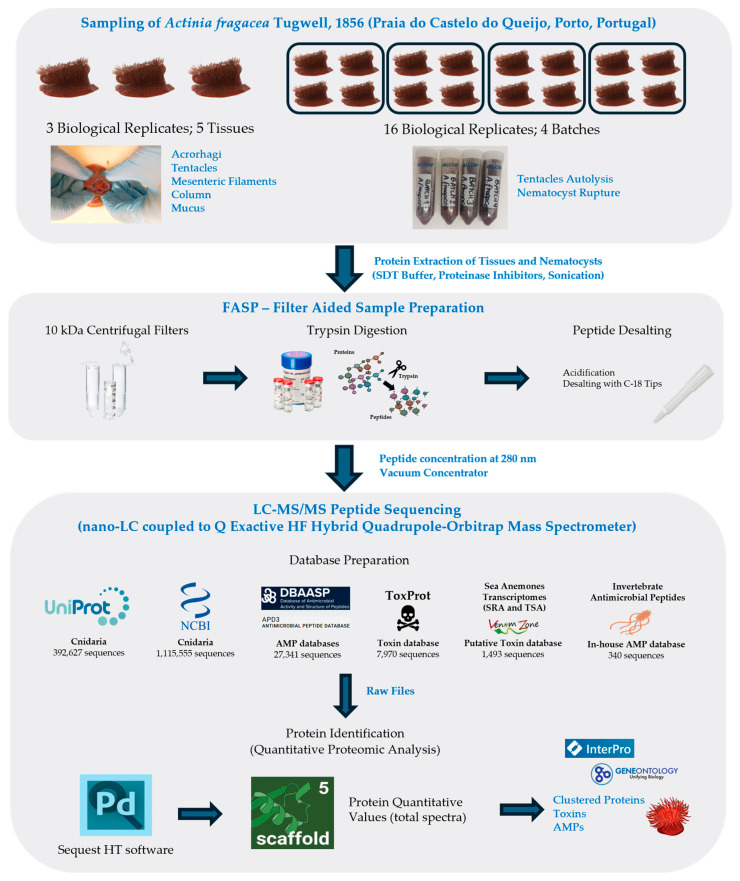
Methodological workflow for proteomic profiling of nematocysts and tissues in *A. fragacea*.

Recent advances in omics technologies—including genomics, transcriptomics, proteomics, and metabolomics—have significantly deepened our understanding of marine organisms’ ecology, distribution, and defense mechanisms and the ability to uncover novel bioactive compounds for biotechnological applications. Despite these developments, the chemical arsenal and protein composition of *A. fragacea* remains poorly characterized, with only 111 proteins currently documented in the NCBI database. In this study, we present a comprehensive proteomic profile of the nematocyst venom extract, four different tissues, and the mucus secretion of the sea anemone *A. fragacea*, addressing this knowledge gap (Figure 1). To the best of our knowledge, this research represents the first proteomic characterization of this species.

## 2. Results

### 2.1. LC-MS/MS Analyses and Protein Identification

**Figure 2:**
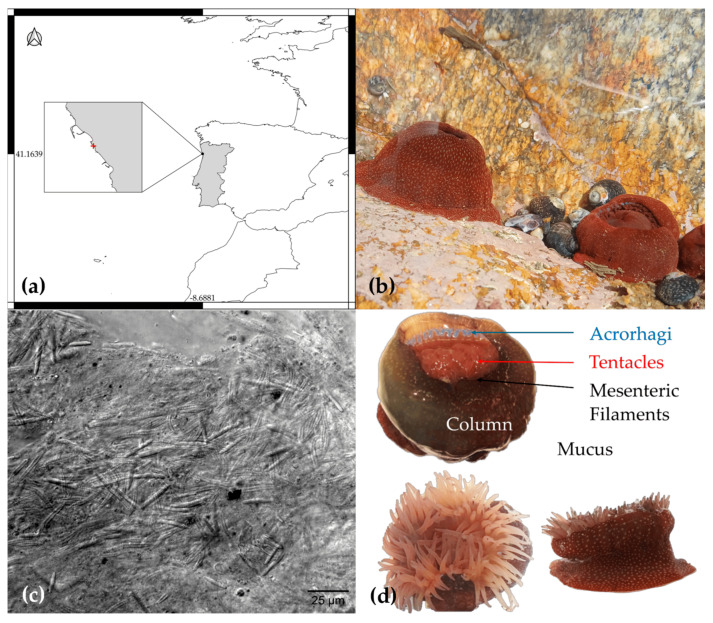
Overview of sampling location, specimens, and experimental approach for *A. fragacea*. (**a**) Map showing the geographic context of the sampling location, centered on Praia do Castelo do Queijo, Porto, Portugal. (**b**) Photograph of *A. fragacea* in its natural habitat, a tide pool at the sampling site, showcasing the species’ distinctive spots in the column. (**c**) Microscopic image of nematocysts isolated from *A. fragacea* tentacles, with a scale bar of 25 μm. (**d**) Photographs illustrating the location of the dissected tissues of *A. fragacea*, including top and side views of the specimen.

Specimens of *A. fragacea* collected from Praia do Castelo do Queijo, Porto, Portugal, were analyzed to assess their protein and peptide content. This study encompassed the nematocyst venom extract, four different tissues, and the mucus secretion, providing a comprehensive overview of the species’ proteomic profile (Figure 2).

A total of 4011 different proteins, clustered into 3383 protein groups, were identified in *A. fragacea* across all analyzed samples (Table S1). Detailed sample composition statistics are presented in Table 1.

**Figure 3:**
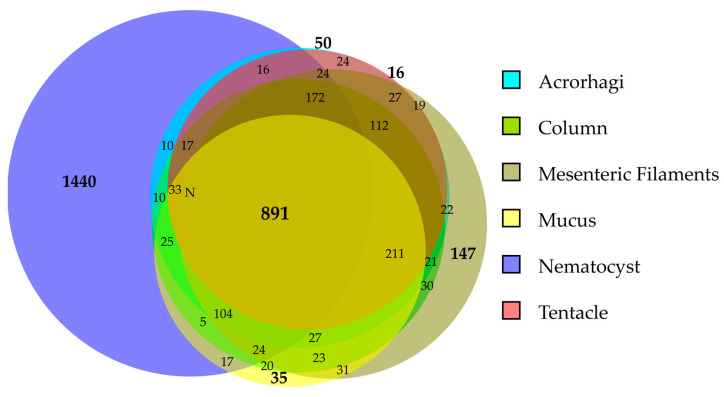
Area-proportional Venn diagram illustrating the overlap of identified proteins across six sample categories, including nematocysts, tissues, and mucus secretions of *A. fragacea*. Generated using DeepVenn (https://www.deepvenn.com, accessed on 10 January 2025).

In total, 891 proteins were ubiquitous and identified across all sample categories (Figure 3). Nematocysts were the samples with more exclusively identified proteins (1440), followed by mesenteric filaments (147), acrorhagi (50), mucus (35), and tentacles (16). Interestingly, no proteins were exclusive from column samples. Acrorhagi, column, and mesenteric filaments shared the highest number of proteins (1735), while mucus, nematocysts, and tentacles shared the lowest (Table 2).

### 2.2. Functional Annotation

**Figure 4:**
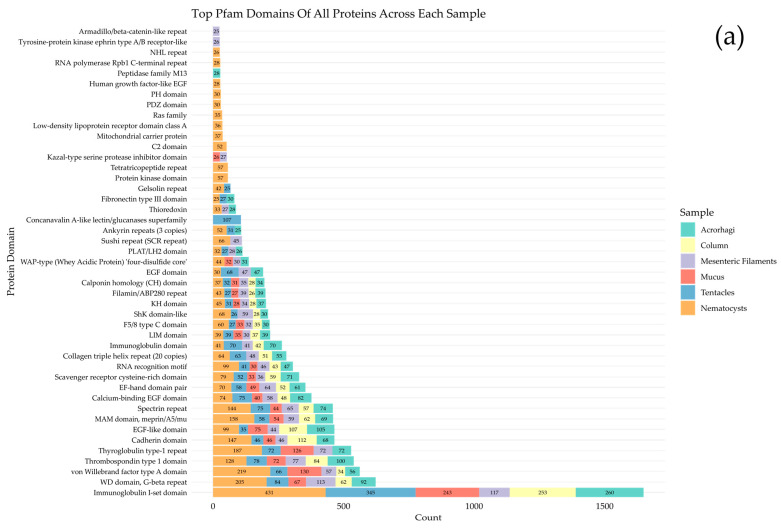

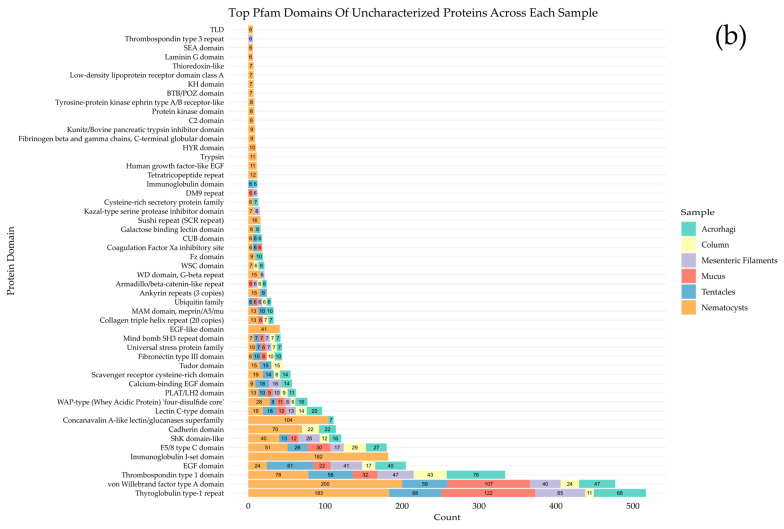
Top Pfam domains identified in *A. fragacea* samples: (**a**) all proteins (minimum 25 copies), (**b**) uncharacterized proteins (minimum six copies).

A comprehensive functional analysis was conducted to gain insight into the biological roles of the identified proteins. InterProScan was used to predict Pfam protein domains and key functional sites, with the analysis performed separately for each sample category to identify functional differences. InterProScan was run independently for each sample category to all validated proteins and a subset of uncharacterized proteins (e.g., hypothetical, predicted without annotation, unnamed protein product) (Figure 4). The results revealed that the Immunoglobulin-I set domain is the most abundant across all sample types. Nematocyst samples contained the most identified protein domains, including several exclusively found in them. Interestingly, the Concanavalin A-like lectin/glucanases superfamily was the second most abundant domain in tentacle samples but was absent in all the other categories. Also, the Thyroglobulin type-1 repeat domain was highly present in all sample types except the column. Among uncharacterized proteins, the most common domains across all sample types included Thyroglobulin type-1 repeat, von Willebrand factor type A, Thrombospondin type 1, and EGF domains. These are often associated with protease inhibition, cell adhesion, tissue remodeling, and cell growth. Complete InterProScan results are provided in Table S2.

A common toxic domain found in potassium channel inhibitors of sea anemones, the Stichodactyla toxin (ShK) domain-like, was detected with high abundance across all samples. Among the 17 uncharacterized proteins containing this domain, two proteins from the transcriptomes of *Anthopleura elegantissima* (Aelegantissima_304099_TSA) and *Anemonia viridis* (Avirdis_207505_SRA) identified in nematocysts, showed high percentage identity with zinc metalloproteinases containing ShK and astacin domains from other sea anemones, suggesting potential toxin functionality (Table 3). Additionally, several other major domains associated with the nematocyst proteome that may contain toxic functions include Kazal-type serine protease inhibitor domains, the cysteine-rich secretory protein family (CAP/CRISP), and Kunitz/bovine pancreatic trypsin inhibitor domains. Ubiquitin family domains were detected in all samples, with their peptides potentially possessing immune function. Detailed functional annotation results are available in Tables S2 and S3.

**Figure 5:**
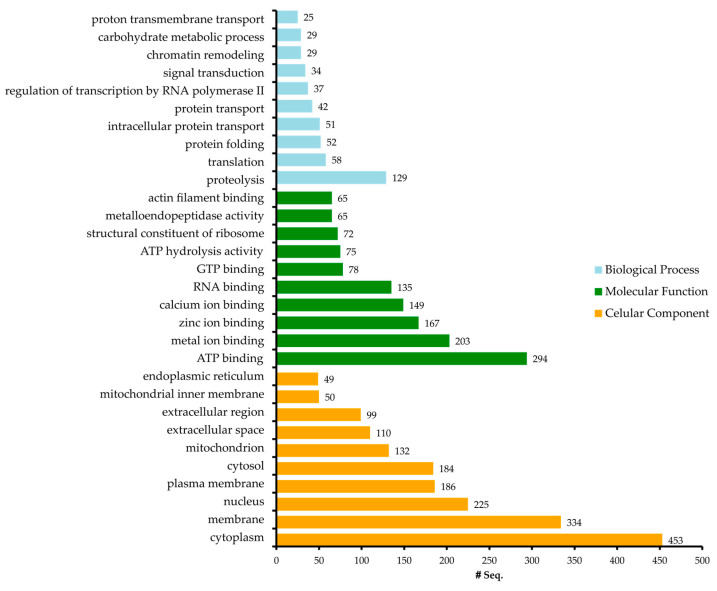
The most represented GO terms of the *A. fragacea* samples in the three domains of biological process, molecular function, and cellular component.

Proteins annotated by InterProScan were further blasted for Gene Ontology (GO) mapping and annotation using Blast2GO software (implemented in the OmicsBox platform version 3.4.0). The annotation process followed standard GO configuration parameters, and redundancy was eliminated through GO validation. Proteins were categorized into the three main GO domains at ontology level 2: cellular component (CC), biological process (BP), and molecular function (MF) (Figure 5). A total of 11,740 GO annotations were assigned, with an average GO level of 6.65 and a standard deviation of 2.39 (Figure S1). The annotation results indicate that the identified proteins are predominantly localized in the cytoplasm, primarily involved in binding functions, and participate in biological processes such as proteolysis.

Additionally, the annotated proteins were categorized according to their enzymatic functions by mapping the corresponding Enzyme Commission (EC) codes to their respective annotations (Figures S2 and S3).

### 2.3. Putative Venom Proteins Identified in A. fragacea

**Figure 6:**
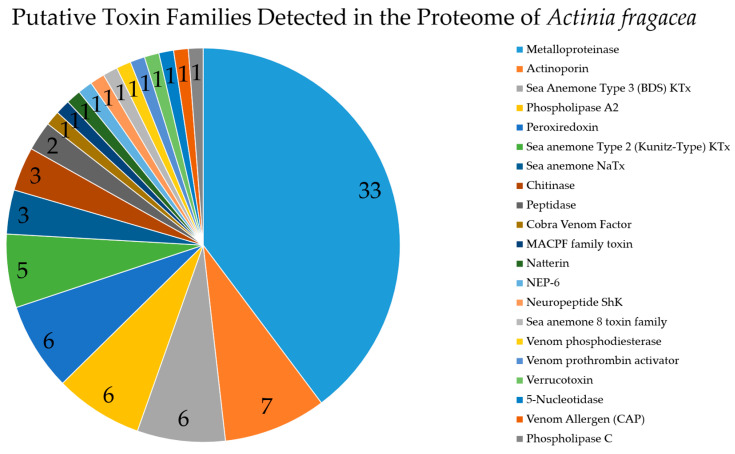
Overview of toxin family types detected in the proteome of the sea anemone *A. fragacea*, including nematocysts, tissues, and mucus secretions. KTx—potassium-channel inhibitory toxins; NaTx—sodium-channel inhibitory toxins. In the legend, toxin families are arranged in descending order of abundance. Comprehensive details of the detected toxins associated with each family can be found in Supplementary Table S4.

**Figure 7:**
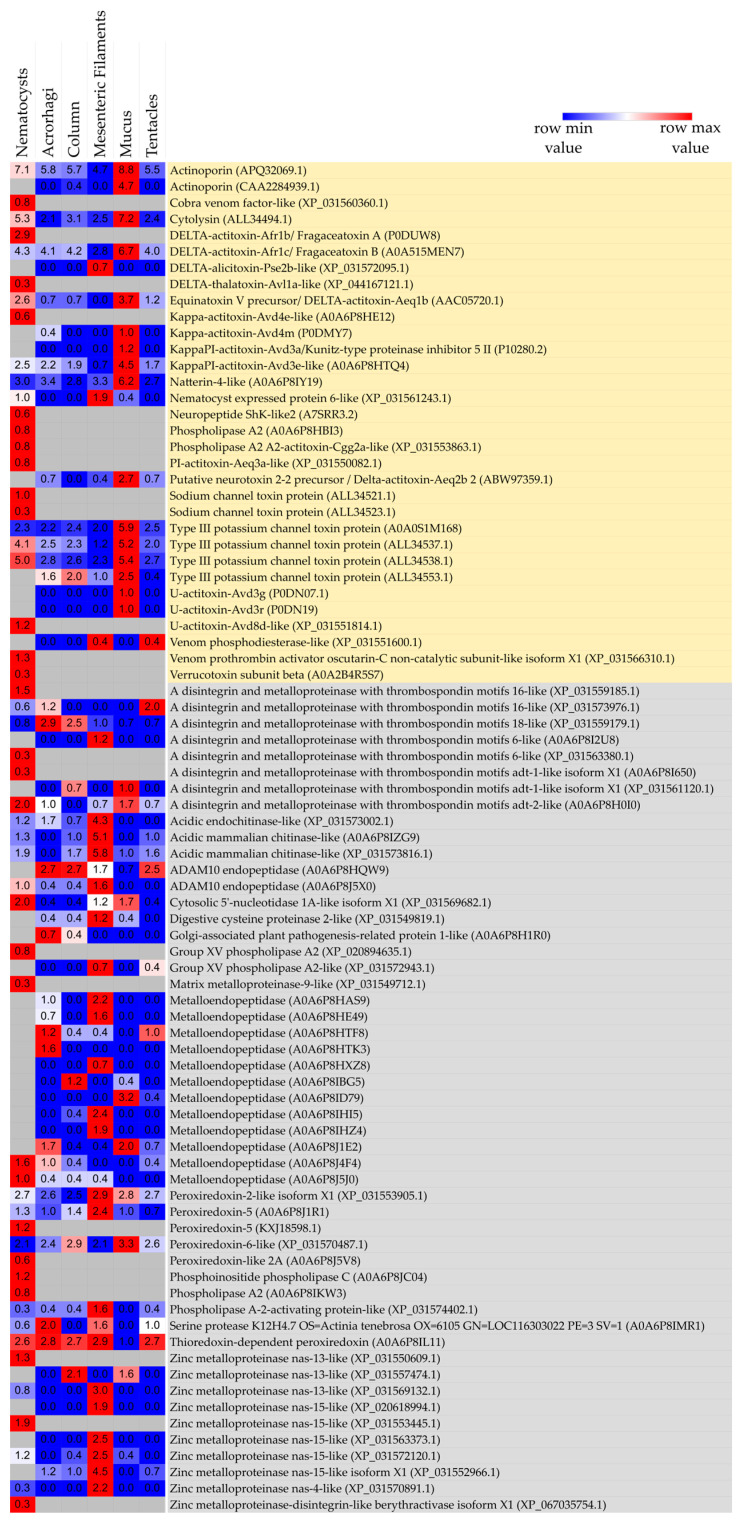
Heatmap of the venom protein expression across nematocysts, tissues, and mucus secretion samples in *A. fragacea*. Proteins highlighted in yellow constitute well-characterized toxins, while those highlighted in blue are related to venom-like functions. The expression levels of each protein across sample types were log-transformed using the formula log_2_ (mean of the normalized total spectrum counts from the three biological replicates +1). The heatmap was constructed using Morpheus https://software.broadinstitute.org/morpheus (accessed on 7 January 2025).

Overall, 83 putative toxins were detected (Figure 6). Of these, 32 constitute well-characterized toxins, while 51 may be related to venom-like functions (Figure 7).

**Figure 8:**
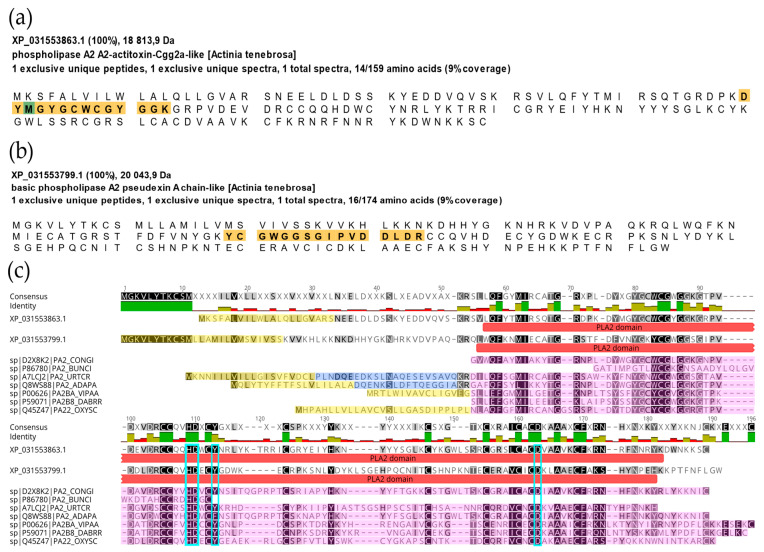
Identification and alignment of the phospholipase A2 (PLA2). The amino acid sequence of the two identified PLA2 domains in the nematocyst proteome of *A. fragacea* are represented in (**a**) XP_031553863.1 and (**b**) XP_031553799.1, both derived from the genome of *A. tenebrosa*. (**c**) Multiple sequence alignment of the identified PLA2 with others from sea anemones (*Condylactis gigantea* [CONGI], *Bunodosoma caissarum* [BUNCI], *Urticina crassicornis* [URTCR], *Adamsia palliata* [ADAPA] and snakes (*Vipera ammodytes* [VIPAA], *Daboia russelii* [DABRR], and *Oxyuranus scutellatus* [OXYSC]). The signal peptides are highlighted in yellow, the propeptide in dark blue, and the mature peptide in pink. The PLA2 domain detected in the identified proteins is marked in red. The catalytic center characteristic of PLA2 is highlighted in light blue. Generated using Geneious software (version 11.1.5).

Most toxins identified showed close identity with those from *Actinia* (59/83), followed by *Anemonia sulcata* (8/83), *A. equina* (3/83), and *A. viridis* (3/83). Two common actinoporins from *A. fragacea* were detected in the proteomic data: DELTA-actitoxin-Afr1b/Fragaceatoxin A (P0DUW8), which was exclusively expressed in the nematocysts, and DELTA-actitoxin-Afr1c/Fragaceatoxin B (A0A515MEN7), which was expressed in all sample types but exhibited higher expression in the mucus. Among the toxins detected, most were identified as putative metalloproteinases (33/83), followed by actinoporins (7/83), members of the sea anemone type 3 (BDS) potassium channel toxin family (6/83), and phospholipases A2 (6/83). XP_031553863.1 and XP_031553799.1 from *A. tenebrosa* were classified as toxins based on the presence of the phospholipase A2 domain (Table S2), high sequence similarity to other PLA2s from other sea anemones and snakes, and the presence of conserved catalytic residues (Figure 8).

**Figure 9:**
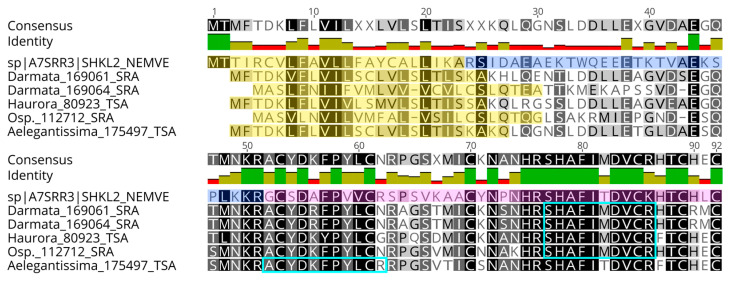
Multiple sequence alignment of ShK-like neuropeptides detected in the nematocyst proteome of *A. fragacea*. These are derived from the transcriptomes of the sea anemones *Dofleinia armata* (Darmata_169061_SRA, Darmata_169064_SRA), *Heteractis aurora* (Haurora_80923_TSA), *Oulactis* sp. (Osp._112712_SRA), and *Anthopleura elegantissima* (Aelegantissima_175497_TSA) and had high sequence identity (E-value 1 × 10^−13^–4 × 10^−10^) with the neuropeptide Shk-like2 from the sea anemone *N. vectensis* (A7SRR3). The signal peptides are highlighted in yellow, the propeptide in dark blue, and the mature peptide in pink. The peptides identified in the proteome of *A. fragacea* are highlighted in light blue. Generated using Geneious software (version 11.1.5).

Other neurotoxins commonly found in sea anemones were also detected, including five sea anemone type-2 potassium channel toxins/Kunitz-type proteinase inhibitors, two sea anemone sodium-channel inhibitory toxin families, and one sea anemone 8 toxin family. Other interesting compounds include the detection of Natterin-4-like expressed in all sample categories but with higher expression in the mucus and the Verrucotoxin subunit beta, detected exclusively in nematocysts. These are common toxins from the venom glands of *Thalassophryne nattereri* and *Synanceia verrucosa* fish. Furthermore, two toxins typically found in the sea anemone *Nematostella vectensis* were identified: the metalloprotease nematocyst expressed protein 6-like, present in all tissues, with higher expression in the column, and the neuropeptide ShK-like2, which was detected exclusively in the nematocysts (Figure 9). All information is available in Table S4.

**Figure 10:**
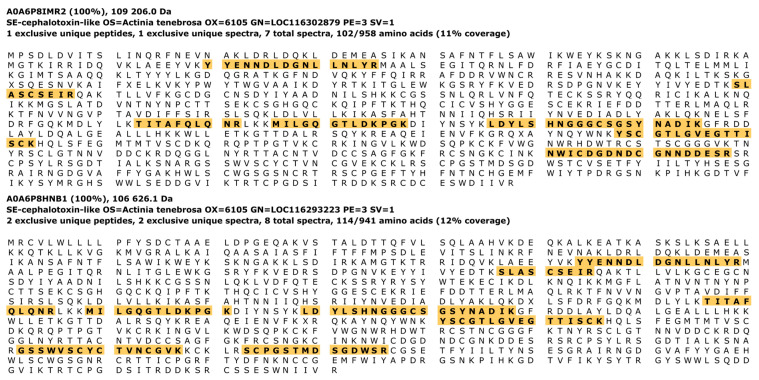
Amino acid sequences of the two identified cephalotoxin-like proteins, with the detected peptides highlighted.

In addition, a whole animal sample was included as a preliminary test, and interestingly, this analysis revealed the presence of two exclusively identified cephalotoxin-like proteins, which are known for their neurotoxic properties and potential bioactivity (Figure 10).

### 2.4. Putative Peptides with Antimicrobial Activity Identified in A. fragacea

**Figure 11:**
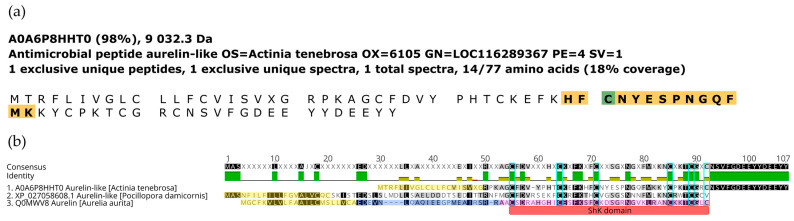
Identification and alignment of the Aurelin-like antimicrobial peptide. (**a**) Amino acid sequence of the identified Aurelin-like antimicrobial peptide, with peptides detected in this study highlighted. (**b**) Multiple sequence alignment of Aurelin from *A. aurita* and Aurelin-like antimicrobial peptides from *A. tenebrosa* and *P. damicornis*. The signal peptides are highlighted in yellow, the propeptide in dark blue, and the mature peptide in pink. The ShK domain is marked in red, while the six cysteine residues forming the conserved motif are highlighted in light blue. Generated using Geneious software (version 11.1.5).

Several bioactive compounds with potential immune-related and antimicrobial functions were identified, providing valuable insights into the organism’s defense mechanisms. Among these, AMPs such as Aurelin-like peptide and Myticin-A were detected (Figure 11). Additionally, immune-related molecules, including neuropeptides like RFamide, enzymes such as peroxiredoxins, and PRPs like lectins were also detected (Table S5). These findings highlight the organism’s diverse biochemical repertoire for pathogen defense and immune regulation.

To further explore the antimicrobial potential of the identified trypsinized peptides, peptide predictions were performed using the Collection of Anti-microbial Peptides (CAMPR4) https://camp.bicnirrh.res.in/ (accessed on 20 January 2025), and the Antimicrobial Peptide Scanner vr.2 https://www.dveltri.com/ascan/v2/ascan.html (accessed on 20 January 2025). The CAMPR4 tool predicted a total of 2950 AMPs, while the Antimicrobial Peptide Scanner vr.2 identified 2073 with antimicrobial potential (Table S6). Upon comparison, 1037 peptides were predicted as AMPs by both tools, which represents approximately 5% of the 20,353 distinct peptides identified in the entire dataset. Importantly, only nine peptides from the total dataset achieved a prediction probability greater than 90% in both tools, indicating a relatively high level of confidence in their antimicrobial potential (Table 4).

## 3. Discussion

### 3.1. Comparative Analysis of Protein Content and Distribution Across Sample Categories

Total protein content varied significantly across the different sample categories. Nematocysts exhibited the highest protein content, followed by mesenteric filaments and acrorhagi, while mucus samples showed the lowest. These differences may reflect the distinct nature of the samples and variations in the sample preparation protocols. Notably, nematocyst samples were processed using a different method than the other sample types, as explained in the Section 4. The standard deviation for proteins and protein groups was particularly high in the mucus samples, potentially reflecting the inherent variability of mucus composition or challenges associated with sample collection. Nematocyst samples were the samples with the most distinctive content. Internal tissues (mesenteric filaments and column) show stronger overlap, likely due to shared metabolic or structural proteins, whereas mucus and nematocyst samples have distinct protein profiles due to their more specialized functions.

### 3.2. Functional Annotation of Identified Proteins

The most common Pfam domains identified in the proteome of *A. fragacea* were associated with housekeeping functions. Immunoglobulin I-set domains, which are involved in immune defense mechanisms in antibodies or cell surface receptors, were also receptors. However, like other marine invertebrates, sea anemones lack adaptive immune responses and instead rely on their innate immune systems, including PRRs—pattern recognition receptors—that recognize intracellular and extracellular molecular patterns associated with microorganisms or cellular damage [36]. The high presence of this domain in the proteome of *A. fragacea* underscores their critical role in survival in high microbial marine environments. Several immunity-related domains were also identified in the immunotranscriptome of other Anthozoans [37]. These domains may be associated with cell adhesion molecules (CAMs), which mediate cell interactions in the nervous system and potentially support nerve regrowth and tissue organization in sea anemones [38]. Additionally, other commonly found domains in the proteome of *A. fragacea* included the von Willebrand A (VWA), Thrombospondin type 1 (TSP1), Thyroglobulin type-1 repeat, and Epidermal Growth Factor (EGF) domains. The VWA domain is known to be involved in cell adhesion, extracellular matrix proteins, and integrin receptors [39]. TSP1 proteins are multidomain glycoproteins involved in tissue homeostasis and remodeling. Notably, the phyla Cnidaria and Porifera encode a broader diversity of TSP superfamily members than vertebrates [40]. For instance, a TSP from *Hydra magnipapillata* was identified as a major component of the cnidarian mesoglea, where it plays a crucial role in head regeneration [41]. Similarly, a TSP from the sea anemone *N. vectensis* was found to be upregulated during regeneration, being present in neuron-like cells within the mesoglea of the retractor muscles and the pharynx [42]. Thyroglobulin type-1 repeat is often associated with protease inhibition. For instance, Equistatin, detected in the mesenteric filaments of *A. fragacea*, is a protein found in *A. equina* that contains three of these domains and acts as an inhibitor of papain-like cysteine proteinases and the aspartic proteinase cathepsin D [43] (Table S4). EGF domains, which are associated with cell growth, proliferation, and differentiation, were also notably abundant. Additionally, an EGF-like toxin, Gigantoxin I (UniProt: Q76CA1), isolated from the nematocysts of the sea anemone *Stichodactyla gigantea*, shows 31-33% sequence identity with mammalian EGF. This toxin exhibits EGF-like activity by inducing cell rounding in human epidermoid carcinoma A431 cells and promoting tyrosine phosphorylation of the EGF receptor while also displaying potent paralytic activity in crabs [44].

Several toxin-like domains were also detected in the proteome of *A. fragacea*. ShK domains were found in mucin-like glycoproteins from nematodes, which are implicated in parasite immune invasion [45], and in cysteine-rich secretory proteins (CRISPs), found in the mammalian male reproductive tract and in the venom of reptiles [46]. In sea anemones, these domains are associated with potassium channel toxin inhibitors, including BgK from *Bunodosoma granulifera* [47], and ShK from *Stichodactyla helianthus* [48]. In the current study, two proteins with zinc metaloproteinases and ShK domains, homologous to other sea anemone metalloproteinases, were detected, suggesting a potential role in toxin function. Similar findings were reported in the jellyfish *Pelagia noctiluca* and the sea anemone *N. vectensis* [49]. Domains commonly associated with protease inhibitors were also detected in the nematocyst proteome of *A. fragacea*, including Kunitz/bovine pancreatic trypsin inhibitor domains, commonly found in other venomous organisms, and Kazal-type serine protease inhibitors, previously detected in the venom of other sea anemones [50].

Additionally, several ubiquitin domains were also identified. While ubiquitin is primarily recognized for its role as a post-translational modification that targets proteins for proteasomal degradation, ubiquitin-derived peptides are known to contain antimicrobial activity [51], making them an interesting target for biomedicine in cnidaria proteomic studies.

### 3.3. Identification of Putative Venom Proteins in A. fragacea Reveals a Diverse Toxin Arsenal

Sea anemones are known to produce a diverse range of toxins with mixed functionalities. To understand their chemical arsenal, several proteomics studies have been made, including for *Bunodactis verrucosa* [52], *B. caissarum* [53], *Bunodosoma cangicum* [54], *Entacmaea quadricolor* [55], *Heteractis magnifica* [56], and *Stichodactyla haddoni* [57].

Here, we detected different toxin types. We detected seven actinoporins, common pore-forming toxins specific to sea anemones that interact with sphingomyelin from animal cell membranes to form oligomeric pores [58]. Here, we detected two out of the five fragaceatoxins previously isolated from *A. fragacea* itself: FraA and FraB. We may have only detected these because they are known to be the most thermally stable isoforms. Additionally, they are the most potent toxins causing hemolysis (HC50 of 0.4 and 0.3, respectively) of sheep red blood cells [34]. Both are expressed in the nematocysts of *A. fragacea*, but curiously, only FraB was detected in the tissues and mucus secretion, with higher expression in mucus, which could mean distinct intracellular locations and ecological functions. Additionally, Equinatoxin-V (DELTA-actitoxin-Aeq1b) from *A. equina* [59] was also detected in all sample types (with higher expression in nematocysts and mucus), as well as four other homologous actinoporins from the sea anemones *A. equina* (APQ32069.1), *Oulactis* sp. (CAA2284939.1), and *A. sulcata* (ALL34494.1) and the stony coral *Acropora millepora* (XP_044167121.1). This provides evidence that the *A. fragacea* membrane-active toxin arsenal may be more diversified.

We also detected some neurotoxins. Of the sea anemone potassium-channel toxin family, we found five toxins of type II (venom Kunitz-type family) and six of type III (BDS) with close identity to others from *A. viridis*, *A. sulcata,* and *A. tenebrosa*. Type-II KTx are homologous to Kunitz-type inhibitors of serine proteases, possessing dual functionality by blockage of Kv activity, namely Kv1.2 channels, and also by inhibiting rapid degradation of the venom by endogenous enzymes of prey [13]. On the other hand, type-III KTx include BDS-I and II from *A. viridis* [60] and APETx1 from *A. elegantissima* [61] and are involved in blocking of Kv3 subunits or ERG (ether-a-go-go, Kv11.1) channels [13]. We also detected two sea anemone sodium-channel toxins, including Delta-actitoxin-Aeq2b 2 from *A. equina*.

Previously, metalloprotease NEP-6 was detected in the nematocytes and pharyngeal glands in later life stages of the sea anemone *N. vectensis* [62]. Here, we had a similar pattern, where a NEP-6-like toxin is expressed in both nematocysts and mesenteric filaments and may be a common toxin expressed in all sea anemones that may aid in digestion. Another interesting compound from *N. vectensis*, the toxin-like Neuropeptide ShK-like2, was also detected in the nematocysts of *A. fragacea*. This neuropeptide, together with the neurotoxin ShK-like1, have a contraction paralysis effect on zebrafish and body contraction on *Nematostella* polyps [63]. As previously mentioned, the ShK-like2 peptide is conserved throughout sea anemone phylogeny [63]. In the present work, homologous genes to this peptide were detected in the transcriptomes of *A. elegantissima*, *D. armata*, *H. aurora*, and *Oulactis* sp., further supporting this assumption.

Additionally, we detected two interesting PLA2 domains (XP_031553863.1 and XP_031553799.1, both derived from the genome of *A. tenebrosa*) with close identity to others from sea anemones and snakes, with the conserved catalytic domain. PLA2 has been convergently recruited into the venom of several taxa, including cephalopods, cnidarians, insects, arachnids, and reptiles [64].

Other interesting toxins found in the proteome of *A. fragacea* included a toxin homologous to the Verrucotoxin subunit beta (A0A2B4R5S7) [65], which has hemolytic, cytolytic, hypotensive, and calcium-channel-modulation activities, and Natterin-4-like (A0A6P8IY19), a class of toxins from stonefish that demonstrates nociceptive, edema-inducing, and kininogenase activities [66]. This is evidence of convergent evolution of toxins across the animal kingdom, and sea anemones may have ancestral toxin-like genes that suffered neofunctionalizations.

### 3.4. A. fragacea Reveals Peptides with Putative Antimicrobial Activity

Sea anemones are known to produce several bioactive compounds that contribute to their innate immune defense system. Among these, AMPs are particularly understudied, with only two examples reported to date: Crassicorin from *U. crassicornis* [29] and Equinin from *A. equina* [67]. In this work, two putative AMPs were identified: Aurelin-like peptide and Myticin. Aurelin, the first AMP described in cnidarians, was initially isolated from the moon jellyfish *A. aurita* [21]; a putative Aurelin homolog was predicted from the transcriptome of the coral *P. damicornis* (XR_003447557), while another potential homolog, referred to as Aurelin-like peptide, was identified in *A. tenebrosa* (A0A6P8HHT0). This peptide has a 6-cysteine residue motif and a ShK domain characteristic of Aurelin. The Aurelin-like peptide identified in *A. fragacea* in the present work may represent another member of a cnidarian-exclusive Aurelin peptide family. Given its evidence in the proteome of a sea anemone, evolutionary studies targeting this family should be performed to understand the origin and phylogenetic distribution of Aurelin-like peptides. Myticin, on the other hand, is a well-characterized AMP that has been exclusively identified in mussels [68]. Its detection in *A. fragacea* samples is likely attributable to cross-contamination, given the strong evidence supporting its mussel-specific origin. This is further supported by the presence of mussels (*Mytilus* spp.) in the tide pools where the sea anemones were collected, which could explain the detection of Myticin in the samples. This finding underscore both the importance of Myticin in *Mytilus* defense system and the need for caution when interpreting AMP data from environmental samples.

Putative peptides with antimicrobial activity include not only AMPs but also other bioactive compounds with antimicrobial properties. Among these are RFamide peptides, a class of neuropeptides defined by a C-terminal arginine and an amidated phenylalanine motif. While their primary function involves neuroregulation, such as cell signaling within the nervous system [69], RFamide III identified in the cnidarian *Hydra* also exhibits antimicrobial activity [70]. In the present work, an Antho-RFamide neuropeptide type-1-like isoform X2 was detected in all sample types analyzed except for the mesenteric filaments. Peroxiredoxins are a conserved family of antioxidant enzymes present across all domains of life that use a highly reactive cysteine residue to neutralize hydroperoxides [71]. Their activity relies on a recycling mechanism mediated by thioredoxin, which restores their catalytic function by reducing oxidized cysteine residues in their active sites. Peroxiredoxins are classified into six subfamilies, four of which are represented in cnidarians (Prx1-AhpC, BCP-PrxQ, Prx5, and Prx6) [72]. A key portion of the (Prx5) peroxiredoxin-5 active site (**AFTPGCSK**THL) has been identified. Moreover, peroxiredoxins also perform functions such as molecular chaperone and phospholipase activities, contributing to the host antimicrobial defenses.

The innate immune response is mediated by the recognition of pathogen-associated molecular patterns (PAMPs) through pattern recognition proteins (PRPs). Lectins, which are carbohydrate-binding proteins, play essential roles in immune responses, cell signaling, and pathogen recognition [73]. By binding to PAMPs such as lipopolysaccharides in Gram-negative bacteria or mannose in fungi, certain lectins can trigger innate immune responses, including processes like phagocytosis and the activation of signaling pathways involved in inflammation and immune defense [74]. In this study, Galectin, L-rhamnose-binding lectin ELEL-1-like, and Malectin-A-like lectins were directly identified from the Scaffold report, while C-type lectin domain-containing protein and SUEL-type lectin domain-containing protein were detected through GO annotations. Additionally, one uncharacterized protein was manually identified based on its GG-type lectin domain profile, further emphasizing the diverse lectin repertoire present in *A. fragacea*. Additional proteins associated with pattern recognition processes were identified, including Toll-interacting protein-like, and a variety of scavenger receptors (SRs). SRs represent a highly diverse superfamily of innate immunity genes that are essential for microbial ligands recognition and facilitating phagocytosis of pathogens [75]. Among the identified SRs were macrophage SR types I and II-like, SR cysteine-rich type-1 protein M130 and M130-like, and SR cysteine-rich domain superfamily protein-like.

## 4. Materials and Methods

### 4.1. Sample Collection and Preparation

A total of 19 specimens of the sea anemone *A. fragacea* were collected in April 2024 from Praia do Castelo do Queijo, Porto, Portugal (41.1639341, −8.6880545) during low tide (Figure 2). The specimens were maintained in aerated aquaria under starvation conditions for two days to eliminate potential impurities, such as food particles and excretions. The samples were divided into two groups. The first group consisted of four batches, each containing four biological replicates (*n* = 16). Tentacles were dissected from the specimens and placed in distilled water at 4 °C to induce osmotic lysis of nematocytes, leading to the release of the nematocysts. The resulting suspension was filtered using Falcon cell strainers with a 100 μm mesh size and centrifuged at 10,000× *g* for 15 min at 4 °C to remove debris and concentrate the nematocysts. The isolation of nematocysts was confirmed through electron microscopy using a Leica DMi8 widefield microscope equipped with a Hamamatsu ORCA Flash v3 camera, and the samples were stored at –80 °C until protein extraction. The second group comprised three specimens (*n* = 3). Mucus and four tissue types—acrorhagi, tentacles, mesenteric filaments, and column—were dissected and stored at –80 °C for subsequent analyses.

### 4.2. Protein Extraction

Nematocyst and tissue samples were thawed on ice and incubated in SDT buffer (2% SDS, 0.1 M Tris/HCl pH 7.6, and 0.1 M dithiothreitol) at a ratio of 0.5 g fresh weight/mL. Protease inhibitors (Halt PI Cocktail, Cat No. 78429, Thermo Fisher Scientific, Waltham, MA, USA) were added at a 1:100 dilution. Samples were kept in the dark at room temperature (RT, 21 °C) for 20 min. Tissue samples were homogenized for 1 min in a cold room (0 °C) using a benchtop homogenizer (Kinematica Polytron Model PT MR 2100). All samples were sonicated for 1 min, with 10 s intervals, at a pulse amplitude of 15 µm (MSE Soniprep 150). Samples were heated to 95 °C for 3 min, followed by centrifugation at 16,000× *g* for 1 h at RT. The resulting supernatants were transferred to new Eppendorf tubes, and total protein concentration was measured by absorbance at 280 nm using a NanoDrop One spectrophotometer (Thermo Fisher Scientific, Waltham, MA, USA). Extracted protein samples were stored at –20 °C until further use.

### 4.3. Filter-Aided Sample Preparation for LC-MS Analyses

The Filter-Aided Sample Preparation (FASP) method was performed as described by Wisniewski et al. (2009) [76] with modifications. Protein samples were diluted to a concentration of 1 μg/μL, in SDT buffer. A total of 30 μL of diluted protein sample was mixed with 200 μL of UA buffer (8 M urea in 0.1 M Tris/HCl, pH 8.5). The mixture was transferred to pre-washed filter units (Merck Millipore Amicon Ultra 0.5 mL, Ultracel 10 K, Cat. No. UFC501096, Merck Millipore, Darmstadt, Germany) and centrifuged at 14,000× *g* for 20 min. The flow-through was discarded, and iodoacetamide solution (100 μL, 0.05 M iodoacetamide in UA buffer) was added to the filter, mixed at 600 rpm for 1 min using a Thermomixer, and incubated in the dark at RT for 20 min. Filters were then centrifuged at 14,000× *g* for 20 min. Three washes were performed with 100 µL UA buffer for sample washing, centrifuging at 14,000× *g* for 15 min each. Three additional washes were carried out using 100 µL of 0.05 M ammonium bicarbonate, centrifuging at 14,000× *g* for 10 min each. Peptide digestion was performed by adding trypsin (Roche, recombinant, proteomics grade, Cat No. 3708985001, Mannheim, Germany) at a 1:100 enzyme-to-protein ratio in 0.05 M ammonium bicarbonate. Samples were mixed at 600 rpm for 1 min in a Thermomixer and incubated in a wet chamber at 37 °C for 16 h. Digested peptides were eluted into new collection tubes by centrifugation at 14,000× *g* for 10 min. A second elution step was performed using 0.5 M NaCl, followed by centrifugation at 14,000× *g* for 10 min. Peptides were acidified with trifluoroacetic acid (TFA, 10% *v*/*v*) to achieve a pH between 2 and 3. For peptide desalting, C18 columns (Thermo Fisher Scientific Pierce C18 Tips, 100 µL, Cat. No. 87784, Thermo Fisher Scientific, Waltham, MA, USA) were conditioned with 80% acetonitrile (*v*/*v*) and 0.1% formic acid (*v*/*v*). Acidified samples were loaded onto the columns and washed with 0.1% formic acid (*v*/*v*) and eluted with 80% acetonitrile (*v*/*v*) and 0.1% formic acid (*v*/*v*) into new tubes. Peptide concentration was measured at 280 nm, and samples were dried using a vacuum concentrator (Eppendorf Concentrator Plus, Eppendorf, Hamburg, Germany). Dried peptides were stored at −20 °C until further processing.

### 4.4. LC-MS Analysis of Protein Samples

The LC-MS/MS was carried out using a nano-LC coupled to Q Exactive HF Hybrid Quadrupole-Orbitrap Mass Spectrometer (Thermo Fisher Scientific, Waltham, MA, USA). Peptide separation was performed by reverse-phase chromatography using an EASY-Spray C18 reversed-phase nano-LC column (PepMap RSLC C18, 2 µm, 100 A 75 µm × 25 cm, Thermo Fisher Scientific) with a gradient of 0.1% formic acid in water (A) and 0.1% formic acid in 80% acetonitrile (B) as follows: from 6% B to 30% B in 65 min; from 30% B to 100% B in 20 min; and 100% B from 85 to 90 min at a flow rate of 0.3 µL/min. Separated peptides were electrosprayed and analyzed using a Q Exactive HF mass spectrometer (Thermo Fisher Scientific, Waltham, MA, USA), operated in positive polarity in a data-dependent mode. Full scans were performed at 120,000 resolutions at a range of 380–1400 *m*/*z*. The top 15 most intense multiple charged ions were isolated (1.2 *m*/*z* isolation window) and fragmented at a resolution of 30,000 with a dynamic exclusion of 30.0 s.

### 4.5. Protein Identification and Quantification

The generated RAW files were analyzed using Sequest HT software package in Proteome Discoverer (v. 2.4.0.305, Thermo Fisher Scientific, San Jose, CA, USA). The Sequest HT search to identify proteins in *A. fragacea* was conducted against a custom database that integrated protein sequences from multiple sources: Cnidaria sequences retrieved from The National Center for Biotechnology Information (NCBI) (1,115,555 sequences, accessed on 13 December 2024) [77], Cnidaria sequences from the Universal Protein knowledgebase (UniProt) (392,627 sequences, accessed on 13 December 2024) [78], sequences from Database of Antimicrobial Activity and Structure of Peptides (DBAASP) (22,272 sequences, accessed on 13 December 2024) [79], sequences from the animal toxin annotation project (ToxProt) (7970 sequences, accessed on 13 December 2024) [80], sequences from the Antimicrobial Peptide Database (APD3) (5069 sequences, accessed on 13 December 2024) [81], putative toxin sequences identified through Hidden Markov Models of cnidarian toxin families from VenomZone available online https://venomzone.expasy.org/ (accessed on 13 December 2024), BLASTp searches against ToxProt on sea anemones transcriptomes (1493 sequences) [82], and sequences from invertebrate antimicrobial peptides (340 sequences) [83].

Sequest was configured to use a fragment ion mass tolerance of 0.1 Da and a parent ion tolerance of 10 ppm; trypsin was selected for protein cleavage, allowing for one missed cleavage. Carbamidomethylation of cysteine was specified as a fixed modification, and oxidation of methionine was specified as a variable modification. MS/MS-based peptide and protein identifications were validated using Scaffold (version Scaffold_5.3.3, Proteome Software Inc., Portland, OR, USA). Peptides were accepted if they met a 0.1% false discovery rate (FDR) threshold, and proteins were accepted with a 1% FDR threshold and required at least one unique identified peptide. Protein probabilities were assigned using the Protein Prophet algorithm [84]. Peptide quantification was based on quantitative values (total spectra). Proteins with similar peptide evidence that could not be distinguished based solely on MS/MS data were grouped to satisfy the principle of parsimony. Only proteins with significant peptide evidence were grouped into clusters and included in further analyses. Venn diagrams were used to identify the shared proteins between different tissues using DeepVenn available online: https://www.deepvenn.com (accessed on 10 January 2025).

### 4.6. Functional Annotation and Expression Profiles

Functional annotation was performed using the BLAST2GO suite [85], implemented through the OmicsBox platform version 3.4.0 available at https://www.biobam.com/omicsbox (accessed on 17 January 2025). This process incorporated InterProScan [86], GO mapping, and GO annotation [85]. Proteins were first categorized into families, and functional domains were predicted using InterProScan. The graphical representation of InterProScan results was generated using R software (version 2024.12.0), *ggplot* package [87]. Potential GO terms were then mapped based on sequence similarity and domain information. Subsequently, GO annotations were refined and validated using experimental data and curated databases, providing a comprehensive characterization of the molecular functions, biological processes, and cellular components associated with the identified proteins.

The expression profiles of putative toxins across different sample categories were visualized using a heatmap, created with the online software Morpheus (Broad Institute, Cambridge, MA, USA, https://software.broadinstitute.org/morpheus, accessed on 14 January 2025). Necessary alignments were performed using Geneious software 11.1.5 (Biomatters Ltd., Auckland, New Zealand; https://www.geneious.com/) (accessed on 19 January 2025).

Additionally, identified peptides were scanned for their potential to be antimicrobial using the Collection of Anti-microbial Peptides (CAMPR4) [88], which employs a Random Forest (RF) algorithm, and the Antimicrobial Peptide Scanner vr.2 [89], which uses a Support Vector Machine (SVM) algorithm. These tools were used to predict peptides from the proteomic dataset that could potentially exhibit antimicrobial activity, based on their sequence characteristics and structural features associated with known AMPs.

## 5. Conclusions

This study represents the first comprehensive proteomic analysis of the sea anemone *A. fragacea*, comparing the protein profiles of its nematocysts, mucus, and tissues. A total of 4011 proteins were identified in the whole proteome, providing a valuable reference for further research on sea anemone biology. Functional bioinformatic analysis revealed that these proteins play key roles in immune responses, cell adhesion, protease inhibition, and tissue regeneration, highlighting the complexity of the proteome. Moreover, some potential toxins were identified, including neurotoxins, membrane-active actinoporins, PLA2 enzymes similar to those found in other sea anemones and snakes, metalloproteinases with ShK domains, and putative cephalotoxins—emphasizing the intricate venom composition used for both prey capture and defense. The study also uncovered AMPs, including an Aurelin-like peptide and nine peptides with predicted strong antimicrobial potential, suggesting an important role in pathogen defense. Additionally, immune-related molecules, such as PRPs, antioxidants, and neuropeptides, were detected. Despite the limitations of not having a transcriptomic dataset for the analyzed specimens, we ensured the accuracy of the results by using a robust cnidarian-specific database, which included sea anemones from the same genus and other closely related species, providing a reliable foundation for our analysis. Our findings provide new insights into the survival strategies of *A. fragacea* and identify bioactive compounds that could have promising applications in biomedical research.

## Figures and Tables

**Table 1:** Summary of results from *A. fragacea* protein profiling experiments by LC-MS/MS.

Sample Category	Proteins (Mean ± SD)	Protein Groups (Mean ± SD)	Total Proteins	Total Protein Groups
Acrorhagi	1358 ± 275	1086 ± 217	1958	1561
Column	1241 ± 203	966 ± 158	1821	1416
M. Filaments	1523 ± 180	1181 ± 129	2078	1580
Mucus	866 ± 334	637 ± 273	1523	1150
Nematocyst	2156 ± 113	1936 ± 107	3009	2769
Tentacle	1237 ± 186	980 ± 154	1660	1322

“Proteins (mean ± SD)” and “Protein Groups (mean ± SD)” indicate the average number and respective standard deviation of unique proteins and protein groups identified per sample category. “Total Proteins” and “Total Protein Groups” show the cumulative counts of unique proteins and protein groups identified across all samples within each category.

**Table 2:** Pairwise comparison of shared proteins across sample categories, highlighted by gradient coloring (red shades indicate higher protein overlap, while blue shades indicate lower protein overlap).

	A	C	F	M	T	N
**A**		1735	1715	1424	1616	1398
**C**	1735		1714	1446	1556	1346
**F**	1715	1714		1446	1574	1407
**M**	1424	1446	1446		1254	1203
**T**	1616	1556	1574	1254		1232
**N**	1398	1346	1407	1203	1232	

A: Acrorhagi; C: Column; F: Mesenteric filaments; M: Mucus; T: Tentacle; N: Nematocysts.

**Table 3:** Identification of a zinc-metalloproteinase (ZnMc) domain and two ShK domains in two proteins identified in the nematocyst proteome of *A. fragacea*.

Protein	Aelegantissima_304099_TSA	Avirdis_207505_SRA
Domain 1	ZnMc (152–291; 5.88 × 10^−52^)	ZnMc (163–302; 2.19 × 10^−51^)
Domain 2	ShK (351–391; 1.96 × 10^−3^)	ShK (364–402; 2.16 × 10^−5^)
Domain 3	ShK (397–432; 6.46 × 10^−4^)	ShK (408–444; 5.24 × 10^−4^)
BLASTp Results	VFU02998.1 (0; 73.77%) XP_031553445.1 (0; 72.31%) VFU02997.1 (2.0 × 10^−160^; 57.89%)	VFU02998.1 (0; 72.65%) XP_031553445.1 (0; 72.54%) VFU02997.1 (2.0 × 10^−150^; 63.88%)
SMART Domains	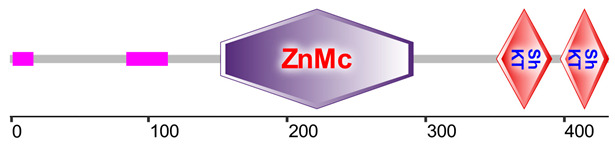	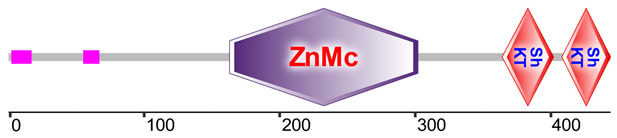

These proteins are homologous to transcripts from *A. elegantissima* and *A. viridis* obtained from the putative toxin-tailored database. The homologous sequences include VFU02998.1 (mRNA, *Oulactis* sp. MM-2018), XP_031553445.1 (zinc metalloproteinase nas-15-like, *Actinia tenebrosa*), and VFU02997.1 (mRNA, *Oulactis* sp. MM-2018). Domain start–end positions and E-values are shown in parentheses. BLASTp results show homologous sequences, along with their E-values and percentage identities. Protein domain representations were obtained using the SMART database [35].

**Table 4:** Peptides predicted to be AMPs with over 90% probability according to both CAMPR4 and Antimicrobial Peptide Scanner vr.2.

ID	Sequence	Protein Name	Accessions	Domains	GO Terms
1280	ASLGQWPAGSYCILASGGCPK	Uncharacterized protein LOC116288220 isoform X2	XP_031550846.1 XP_031550847.1	Periplasmic Binding Protein Type-2 (IPR010068) C-terminal domain of apextrin (IPR031569)	
6378	GIAFPTCISVNNCVCHCSPLK	Proliferation-associated protein 2G4-like	XP_031550715.1	Metallopeptidase family M24 (IPR000994)	
6420	GILFAVPGAFTPGCSK	Peroxiredoxin-5	A0A6P8J1R1	Thioredoxin (IPR013766)	GO:0034599 GO:0016491 GO:0008379
6581	GLGPSVISAVIPLYGK	Uncharacterized protein LOC116295095	A0A6P8I1A2	GG-type lectin domain profile	GO:0005515
7224	GVVIIGPATVGGIKPGCFK	ATP-citrate synthase	XP_031568246.1	Citrate synthase Integrated (IPR036969) Succinyl-CoA synthetase domains Integrated (IPR016102)	GO:0006085 GO:0006101 GO:0003824 GO:0046912 GO:0003878
8437	ILGLPGFSVPPAAFVPVCK	Uncharacterized protein LOC116307005	A0A6P8J4T9	Thyroglobulin_1 (IPR000716)	GO:0030414 GO:0005515 GO:0004867
17784	VCLLGCGISTGYGAAINTAK	S-(hydroxymethyl)glutathione dehydrogenase	A0A8B7E018 T2MHJ5 XP_065652493.1	Class III alcohol dehydrogenases (IPR014183)	GO:0046294 GO:0008270 GO:0051903 GO:0016491
18406	VLDALFPCVQGGTTAIPGAFGCGK	H(+)-transporting two-sector ATPase	KAK3738516.1 XP_020913463.1 A0A6P8IB06	P-loop containing nucleotide triphosphate hydrolases (IPR027417)	GO:0046034 GO:1902600 GO:0005524 GO:0046961 GO:0016887 GO:0033180

## Data Availability

The mass spectrometry proteomics data have been deposited in the ProteomeXchange Consortium via the PRoteomics IDEntifications (PRIDE) Archive repository with the dataset identifier PXD060643. Additional data will be made available on request.

## Supplementary Materials

The following supporting information can be downloaded at: https://www.mdpi.com/article/10.3390/md23020079/s1, Figure S1: Distribution of GO levels across biological process, molecular function, and cellular component domains in *A. fragacea* samples; Figure S2: Enzyme Commission (EC) classes of enzymes identified through GO annotation in the *A. fragacea* proteome; Figure S3. Enzyme Commission (EC) classes of hydrolases identified through GO annotation in the *A. fragacea* proteome. Table S1: Comprehensive protein report derived from *A. fragacea* samples; Table S2: InterProScan results report from general and uncharacterized proteins identified in *A. fragacea* proteome; Table S3: GO Annotation results for proteins identified in *A. fragacea* proteome; Table S4: List of putative toxins identified in *A. fragacea* proteome; Table S5: List of putative immune-related proteins identified in *A. fragacea* proteome; Table S6: Predicted AMPs in *A. fragacea* proteome based on CAMPR4 and Antimicrobial Peptide Scanner vr.2 analyses.
