# Supplementary material for: Proteomic Diversity of the Sea Anemone Actinia fragacea: Comparative Analysis of Nematocyst Venom, Mucus, and Tissue-Specific Profiles
Source: Mar Drugs. 2025 Feb 11;23(2):79. doi: 10.3390/md23020079 (PMC11857728; doi:10.3390/md23020079)
Supplement: Supplementary file 1 [file marinedrugs-23-00079-s001.zip › marinedrugs-3471458-supplementary material.pdf]

Supplementary Material

Proteomic Insights into *Actinia fragacea*: Comparative Analysis of Nematocyst Venom and Tissue-Specific Profiles

Ricardo Alexandre Barroso[1,2,†], Tomás Rodrigues[1,2,†], Alexandre Campos[1], Daniela Almeida[4], Francisco A. Guardiola[5], Maria V. Turkina[3] and Agostinho Antunes[1,2,*]

Figure S1. Distribution of GO levels across biological process, molecular function, and cellular component domains in *A. fragacea* samples. (Total Annotations=11,740; Mean Level=6.65; Std. Deviation=2.39).

Figure S2. Enzyme Commission (EC) classes of enzymes identified through GO annotation in the *A. fragacea* proteome.

Figure S3. Enzyme Commission (EC) Classes of hydrolases identified through GO annotation in the *A. fragacea* proteome.

Table S1. Comprehensive protein report derived from *A. fragacea* samples.

Table S2. InterProScan results report from general and uncharacterized proteins identified in *A. fragacea* proteome.

Table S3. GO Annotation results for proteins identified in *A. fragacea* proteome.

Table S4. List of putative toxins identified in *A. fragacea* proteome.

Table S5. List of putative immune related proteins identified in *A. fragacea* proteome.

Table S6. Predicted AMPs in *A. fragacea* proteome based on CAMPR4 and Antimicrobial Peptide Scanner vr.2 analyses.

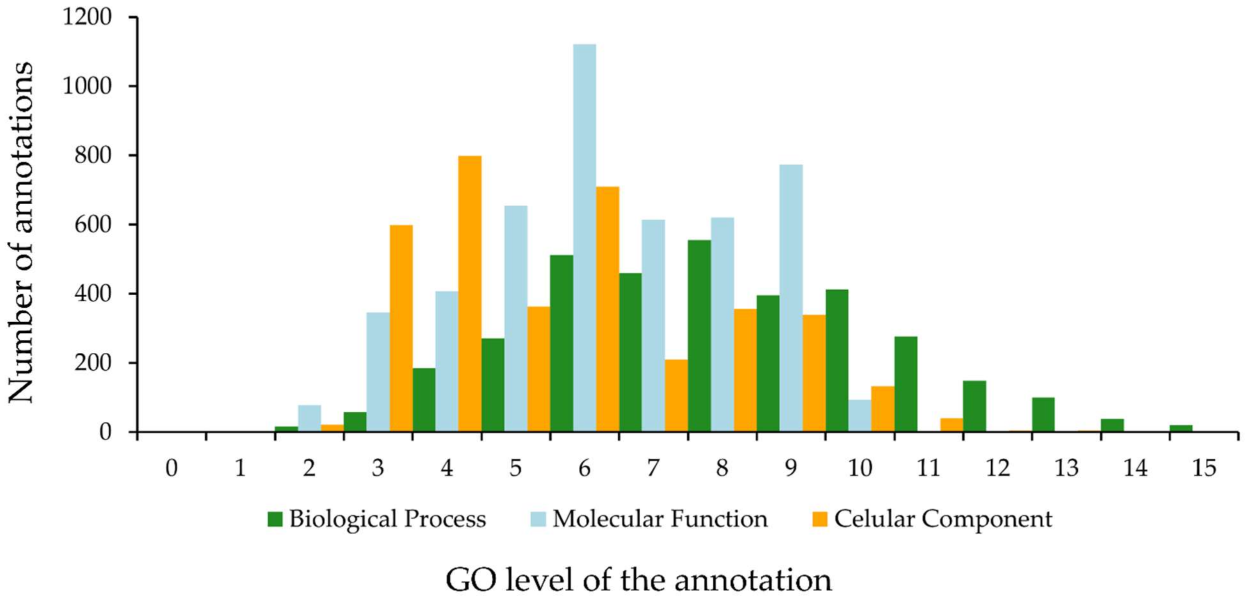

Figure S1. Gene Ontology level distribution across biological process, molecular function, and cellular component domains in *A. fragacea* samples. (Total Annotations=11,740; Mean Level=6.65; Std. Deviation=2.39).

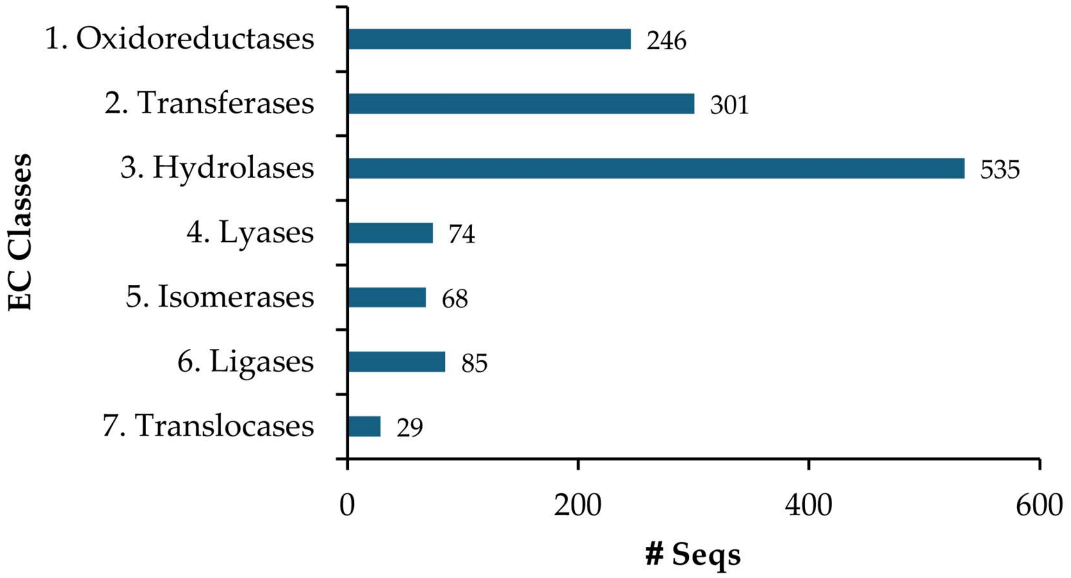

Figure S2. Enzyme Commission (EC) classes of enzymes identified through GO annotation in the *A. fragacea* proteome.

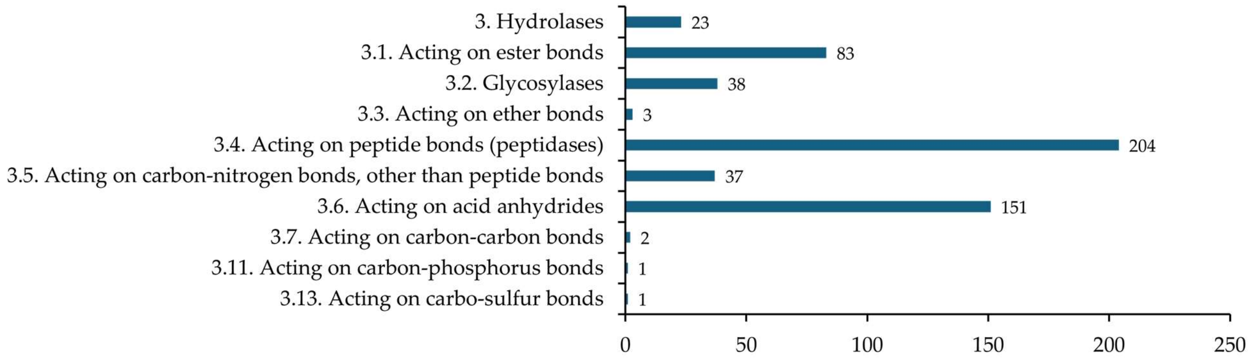

Figure S3. Enzyme Commission (EC) Classes of hydrolases identified through GO annotation in the *A. fragacea* proteome.
